# Urbach Rule in the Red-Shifted Absorption Edge of PET Films Irradiated with Swift Heavy Ions

**DOI:** 10.3390/polym14050923

**Published:** 2022-02-25

**Authors:** Adil Z. Tuleushev, Fiona E. Harrison, Artem L. Kozlovskiy, Maxim V. Zdorovets

**Affiliations:** 1 Flerov Laboratory of Nuclear Reactions, Joint Institute for Nuclear Research, 141980 Dubna, Russia; adilzht@mail.ru; 2 Engineering Profile Laboratory, L.N. Gumilyov Eurasian National University, Nur-Sultan 010008, Kazakhstan; fiona_e_harrison@hotmail.com (F.E.H.); kozlovskiy.a@inp.kz (A.L.K.); 3 Laboratory of Solid-State Physics, The Institute of Nuclear Physics, Almaty 050032, Kazakhstan; 4 Department of Intelligent Information Technologies, Ural Federal University, 620075 Yekaterinburg, Russia

**Keywords:** polyethylene terephthalate, bandgap energy, SHI irradiation, Urbach energy, latent track

## Abstract

This paper presents a new analysis of the experimental transmission spectra of polyethylene terephthalate (PET) films before and after irradiation with swift heavy ions (SHI) films, as reported previously by the authors. It is shown that the absorption edge red shift for irradiated films contains two regions of exponential form, one of which is located in the UV region and the other at lower energy, mainly in the visible part of the spectrum. The behaviour of the transmission curves under different irradiating fluences demonstrates that these two regions reflect respectively the electron-enriched core of the latent track and its electron-depleted peripheral halo. The focal point method yields a bandgap energy of 4.1 eV for the electron-enriched core of the latent track, which is similar to *n*-doped semiconductors, and a bandgap of about 1.3–1.5 eV for the electron-depleted halo, similar to *p*-doped semiconductors. The boundary between the latent track cores and halos corresponds to a conventional semiconductor *p*-*n* junction. The values of the characteristic Urbach energy determined from experimental data correspond to the nonradiative transition energy between the excited singlet and triplet levels of benzene-carboxyl complexes in repeat units of the PET chain molecule. A parallel is drawn between the SHI-induced redistribution of electrons held in structural traps in the PET film and chemical redox reactions, which involve the redistribution of electrons in chemical bonds. It is suggested that alkali etching triggers the release of excess electrons in the latent track cores, which act as a catalyst for the fragmentation of PET chain molecules along the latent tracks of the SHI irradiation.

## 1. Introduction

Irradiation of polymer films with swift heavy ions (SHI) is of ongoing interest [1,2,3], due to the possibilities it offers to modify the properties of films in useful ways. A striking example is the achievement of filtration levels comparable to biological membranes from SHI-irradiated polyethylene terephthalate (PET) films [4,5,6,7]. Optical methods are widely used to investigate the post-irradiation properties of transparent polymer films. One of the most noticeable effects of SHI irradiation is the progressive fluence-dependent red shift of the absorption edge. For high fluences, the broadening of the absorption edge can increase by over an order of magnitude (for example, for PET films, the width of the pristine absorption edge is less than 0.1 eV, but is ~2 eV at high fluences). The absorption edge red shift has been extensively studied [5,8,9,10,11,12,13,14,15,16,17,18,19], and both differential [5,8,9,10,11,14,15,16,17] and integral [12] relationships have been established between the irradiating fluence and the optical transmission/absorption spectra. It is generally agreed that the shift is associated with the formation and subsequent growth of extended *π*-conjugated systems, but there are differing views as to exactly how this growth occurs. Attention has mainly focused on the effects of the fast *δ*-electrons (E ~ 10^3^ eV) excited by the SHIs, and their cascades of secondary electrons [5,8,9,10,11,13,14,15,16,17,18,19]. These studies have concluded that the observed effects are the result of irreversible carbonization of the polymer film due to stochastic destruction of covalent bonds in the polymer chain molecules and subsequent disorderly radiation cross-linking. However, SHI irradiation also excites slow electrons of energies below typical ionisation energies [20]. In [12,21,22,23,24,25], we used optical and X-ray methods to show that the effects of SHI irradiation on PET films depend not only on the energy and fluence of the SHI ions, but also their charge. This charge dependence can only be understood by the consideration of slow electrons. We explained our observation of irradiation-induced ordering and subsequent spiralization of the molecular structure of PET as due to the rotation of the benzene-carboxyl complexes in the PET repeat units (which have a dipole moment) in a residual electric field created by the movement of more numerous slow electrons from the outer periphery into the latent track core to replace those lost through *δ*-electron processes [20]. Electret properties of PET films contribute to the retention of the electron enrichment in the central core of the latent track. A study of the optical spectra of irradiated PET film after thermally stimulated discharge (annealing) showed that electrons were released from the deepest traps (benzene-carboxyl complexes) during thermal relaxation from the irradiation-induced ordered state to a more thermodynamically stable disordered state [12,21].

In the field of electret studies, thermally stimulated discharge/depolarization (TSD) current-thermograms are used to identify various thermal relaxation processes, including the high temperature *α*-relaxation associated with orientational rotations of polar units of macromolecules [26]. The TSD results presented in [27] show clearly that SHI irradiation of PET film leads to an increase in the intensity of *α*-relaxation compared to the pristine film, due, as the authors note, to an increase in orientational rotations of the polar units within the PET molecules. There is only one mobile group with a dipole moment in the PET chain molecule, namely the benzene-carboxyl complex. The results in [27] therefore provide confirmation of our previous conclusions [12,21,22,23,24,25] that the observed effects of induced ordering and spiralization in SHI-irradiated PET films are associated with the orientational movement of benzene-carboxyl complexes in the residual electric field of the cylindrical latent track. We note that our previous paper [25] is misreferenced in [27] as confirming an increase in the number of carboxyl groups as a result of the destruction of PET molecules under the action of SHI irradiation. Our experimental results do indeed show an increased density of carboxyl units on the inner walls of the latent track (without the irradiated film being introduced into water or an aqueous electrolyte), but we do not refer to molecular destruction in our explanation. On the contrary, in our paper we conclude that this increase is the result of the ordering of benzene-carboxyl complexes in the electric field of the latent track.

The question of the relative contribution of destructive and nondestructive processes to the post-irradiation properties of PET films thus remains open. In this paper we address this question through further analysis of the evolution of the shape of the absorption edge under SHI irradiation using experimental optical transmission spectra data published in [12,21,22]. Absorption edges with widths of up to a few eV are observed in a number of amorphous semiconductors, with an exponential dependence of the absorption coefficient *α* on the photon energy *hν* (the Urbach edge) [28]. In [29], SHI irradiation is shown to lead to a broadening of the exponential absorption edge of a thin PbI_2_ film (and other materials) into the red region. The author concluded that this is due to a broadening of the exciton absorption fringe by an internal electric field induced by ion irradiation. The red shift of the Urbach absorption edge with an increase in fluence was studied in [30], when quartz glass was irradiated with Re ions. It was shown in [31,32] that the exponential shape of the absorption edge in semiconductors is caused by the absorption of light in direct exciton transitions in an electric field that is uniform on atomic/molecular scales. According to [33,34], the optical tail of the absorption coefficient *α*(*ν*) in the energy region below the optical bandgap energy *E_g_* is described by the formula

*α*(*ν*) = *α*_0_*exp* − (*E*_*g*_ − *hν*)/*W*, (1)

where *α*_0_ is weakly dependent on *ν* and *W* is a characteristic Urbach energy that does not depend on *ν*. For amorphous materials, *W* is also independent of temperature, and has values in the range 0.05 to 0.2 eV. In [33,34], it was suggested that in an amorphous material, the smooth electric field responsible for the formation of the Urbach edge can be caused by nonlocal structural defects of technological and radiation origin, and that the value of *W* is related to the intensity of the electric field inside the material. Taking these results together, it can be concluded that the experimental signature of both the Urbach edge and the energy range in which excitons exist is a linear dependence between the logarithm of the absorption coefficient *lnα*(*ν*) and the photon energy *hν*:

*lnα*(*ν*) = − (*E*_g_ − *hν*)/*W*. (2)

It is clear, however, that *E_g_* as determined from Equation (2) will underestimate the value of the optical bandgap [28], since *E_g_* is the value of the extrapolation of the straight line *lnα*(*ν*) to the point of intersection with the *ν* axis, and this point lies *below* the energy range of the Urbach edge. Our analysis shows that the parameter *E_g_* from (2) nevertheless provides information about the optical properties of the PET films that we have investigated. In [28] the authors provide a more accurate way of determining the optical bandgap energy from a set of linear *lnα*(*ν*) lines obtained by varying some parameter (e.g., temperature, SHI fluence) that progressively affects the slope of the Urbach edge. The extrapolations of the lines rotate about and converge at a focal point, which indicates a characteristic energy that is constant relative to the variable parameter, as demonstrated in [35] for temperature.

Below we present an analysis of the experimental UV/vis/near IR transmission spectra *T*(*ν*) previously obtained for pristine and SHI-irradiated PET films, and discussed in [12,21,22]. This shows that these spectra also exhibit a linear dependence between the logarithm of the absorption coefficient *lnα*(*ν*) and the photon energy *hν*. We note that the energy balance in a spectrophotometer when measuring the spectral dependence of transmission can be written as 1 *= I_T_ + I_A_ + I_R_ + I_S_*, where the sum on the right respectively comprises the intensities of transmitted, absorbed, reflected and scattered light. In the absorption area, when *α*(*ν*)*d* < 5, and *d* is the thickness of the absorbing layer, the value of *I_S_* << *I_A_* + *I_R_* [36]. The expression becomes 1 = *I_T_* + I_T*_, where I_T*_ = I_A_ + *I_R_* is ‘nontransmittance’. This ‘nontransmittance’ comprises the totality of the interaction between the sample and the electromagnetic disturbance caused by a light wave [37], and is what our spectrophotometer measures when in the ‘absorbance’ mode. We will use the symbol α*(*ν*) to emphasize that it represents the overall attenuation of the intensity of incident light as a result of both absorbance and reflection. Taking into account multiple reflections from internal boundaries, *I_T_* and *I_R_* can also be expressed in terms of the reflection coefficient *R* of the film-air boundary as [36]

*I*_*T*_ = (1 − *R*)^2^*exp*(−*αd*)/[1 − *R*^2^*exp*(−2*αd*)]; *I*_*R*_ = *R*[1 − *I*_*T*_
*exp*(−*αd*)], (3)

from which one can also see that interaction between the incident light and the entire film is involved in both transmission and reflection.

## 2. Experiment

All PET film samples (trade name HOSTAPHAN^®^ polyester films, Mitsubishi Polyester Film Gmbh, 12 μm and 23 μm thick) were irradiated with swift heavy Ar and Kr ions in the DC-60 cyclotron (Nur-Sultan, Kazakhstan). All the ions used in all experiments were of sufficient energy to pass through the PET films. Detailed descriptions of the experimental set-ups and presentation of the observed primary UV/vis/near IR optical spectra have been published in [12,21,22]. Nevertheless, for convenience, we provide here key experimental data from [12,21,22] to enable an understanding of the physical conditions used to obtain the experimental data we discuss here.

## 3. Study of the Dependence of lnα*(*ν*) on *hν*

**Figure 1:**
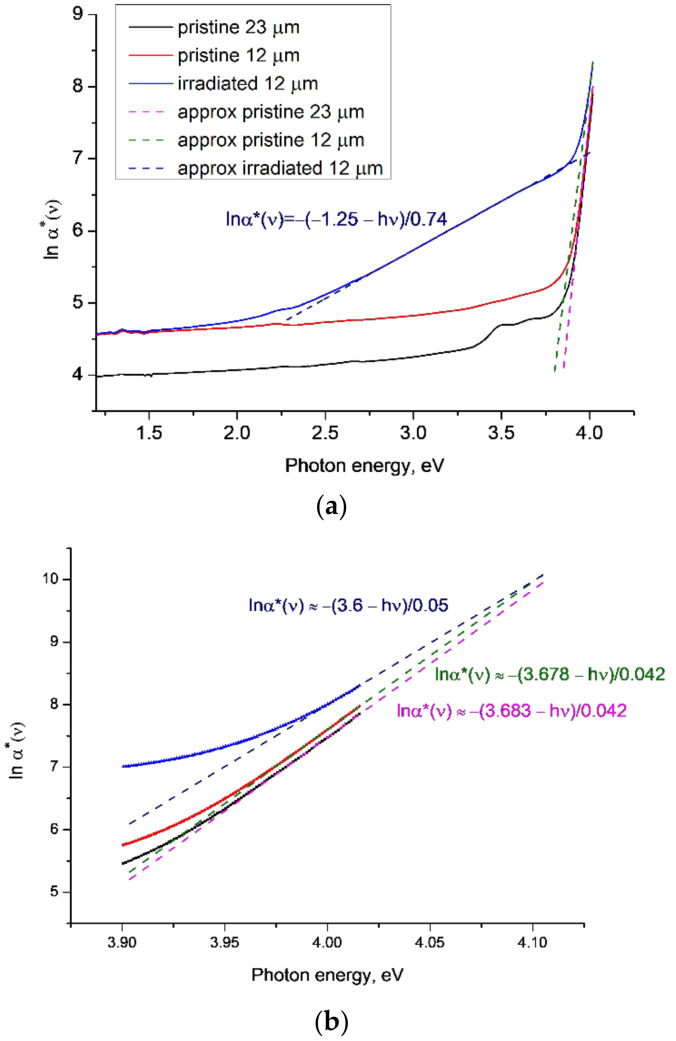
The functions lnα*(*ν*) for experimental results reported in [22] for PET samples irradiated with Kr^15+^ ions of energy 1.75 MeV/a.u. and a fluence of 1.9 × 10^10^ cm^−2^: (**a**) general view; and (**b**) enlarged view of the UV part of the spectra around 4 eV. Dotted lines show linear approximations to the corresponding sections of the experimental plots.

Figure 1 shows lnα*(*ν*) as a function of photon energy *hν* for the high-resolution UV/vis/near IR transmission spectra *T*(*ν*) reported and discussed in [22] for samples of pristine (red) and irradiated (blue) PET film of thickness 12 μm and a pristine sample with a thickness of 23 μm (black line). The other two 12 µm-thick samples reported in [22] have extremely minor differences from the one shown in Figure 1a, so are not included here. The irradiated 12 µm thick sample was irradiated with Kr^15+^ ions of energy 1.75 MeV/a.u. in the normal geometry and a fluence of 1.9 × 10^10^ cm^−2^. We first consider the region of the highest photon energies, shown in greater detail in Figure 1b. For the 12 µm-thick PET film sample, linear extrapolations of the ends of experimental curves for the pristine and irradiated states converge at a focal point at a photon energy of 4.1 eV. This agrees with the experimentally determined [38,39] value of the energy of the singlet transition from the ground *S*_0_ to the excited state *S_1_* in benzene-carboxyl complexes in the repeat units of PET chain molecules. Studies of photo-stimulated discharge spectroscopy of the electret sample of the PET film [40] concluded that it is these benzene-carboxyl groups that form electron traps in the PET film, and thus that the bandgap energy is 4.1 eV. As expected, the parameter *E_g_* is lower than this, at around 3.7 eV and 3.6 eV for the pristine and irradiated films, respectively.

Interestingly, these values of *E_g_* are similar to a value interpreted in [12] as an indicator of the boundary between the core and peripheral halo of a latent track, which was estimated as being about 4nm from the axis of the latent track, in broad agreement with other estimates [3]. The value of *E_g_* for the pristine sample is equal to the energy of the highest triplet state *T*_3_ = 3.7 eV of the benzene-carboxyl complexes found in [39]: the post-irradiation reduction to 3.6 eV indicates, we believe, the broadening of the *T*_3_ line under the influence of the residual latent track electric field. We conclude that the focal point at which these lines converge is the bandgap energy in these circumstances. The values of *E_g_*, as expected, turn out to be lower, but their alignment with the triplet energy level found in [39] does not seem accidental to us, and we intend to investigate this further.

In [31] it is shown that an increase in electric field strength leads to an increase in electroabsorption due to broadening of the exciton line by the electric field. This results in a fan-shaped rotation of the straight lines of lnα*(*ν*) around a focal point as the electric field increases. We have previously shown in [23,24,25] that SHI irradiation results in cylindrical electron redistribution around the latent track axes within PET films, and that the resultant residual electric field is preserved by the electret properties of PET. This suggests that the explanation in [29,31] applies here, with the rotation of the irradiated lnα*(*v*) line in Figure 1b around the focal point of 4.1 eV having an electro-optical nature and being associated with the broadening of the direct exciton transition line by the electric field of the latent track. For the 12 µm-thick PET film, the value of *W* is about 0.04 eV for the pristine and 0.05 eV for the irradiated samples. In line with [34], this suggests that pristine and irradiated PET film material can in some respects be considered as two different materials.

We turn now to the pristine 23 µm-thick sample, whose behaviour at high photon energies was previously indistinguishable from the pristine 12 µm samples [22]. Figure 1b shows two well-separated parallel lines for 12 µm- and 23 µm-thick pristine films, illustrating the sensitivity of this analysis, since in all respects other than thickness, these samples are very similar. The presence of induced gyrotropy in pristine PET film samples [22] and the lower texture levels in thicker samples (confirmed by X-ray analysis in [21]) suggests that the observed small difference in transmission/absorption between the 12 µm and 23 µm samples is related to small variations in the distance between carboxyl groups in repeat units of neighbouring chain molecules.

In [12,23,24,25], we demonstrated the role of carboxyl groups in the formation of a post-irradiation ordered state and the increase in extended *π*-π* conjugated systems with a spiral structure, and showed how this led to the experimentally observed red shift of the absorption edge of PET film after SHI irradiation. We believe that this is an example of the nonradiative Dexter energy transfer mechanism, well known in photochemistry, which decays exponentially with distance [41,42,43]. It results from the overlapping of the electron wave functions of the exciting and excited dipoles, so is very short range (<1 nm) in its action. From [44] and the densities of the amorphous and crystalline parts of PET (1.335 g/cm^3^ and 1.41 g/cm^3^ respectively), we can estimate that the average distance between carboxyl groups during the formation of the helical structures responsible for the induced gyrotropy observed in both pristine and irradiated samples does not exceed 0.7 nm, thus lying within this range. The exponential decay of the Dexter mechanism also provides an explanation for the small but clear difference seen in Figure 1b between the lines for the pristine 12 µm and 23 µm samples.

Returning to Figure 1a, for the irradiated sample there is a second well-defined linear section of lnα*(*ν*) in the energy range 2.7–3.5 eV, indicating that there is a second Urbach edge and region where excitons exist, separated from the higher energy exciton region (around 4 eV) by a transitional region of exciton instability where lnα*(*ν*) is not linear. We believe that these two regions of stable excitons correspond to the core and the halo of latent tracks, with the transitional region corresponding to the boundary between them. This is in line with [33], which found that if the energy of an electric field exceeds the exciton ionisation energy it creates a region of instability of excitons, the boundary of which is determined by the difference between the bandgap energy and the characteristic Urbach energy *W*. The residual electric field due to the electron enrichment/depletion in the core/halo of a latent track [12,21,22,23,24,25] has its greatest radial value at the boundary between the core and halo. We previously found this boundary to be at a radial distance of about 4 nm and in the energy region 3.3–3.6 eV, which is broadly consistent with the end of the lower energy Urbach edge in Figure 1a [12,21]. In this lower energy Urbach edge region *W* ≈ 0.74 eV (we disregard the negative value of *E_g_* for physical reasons as being out of range of our experiment). This value of *W* is much larger than the values given in [34], but we note that it is very close to the energy of the nonradiative transition between *S*_1_ and the triplet level *T*_2_ found in [39].

**Figure 2:**
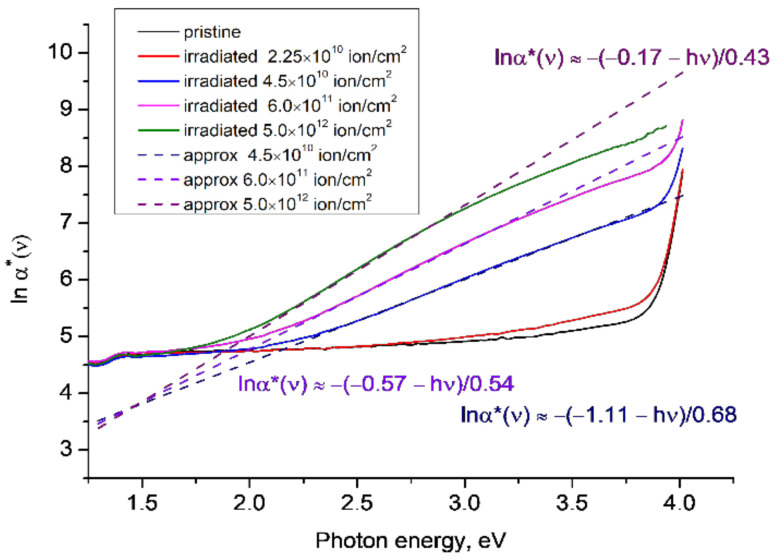
The function lnα*(*ν*) for the experimental results reported in [12] for PET samples irradiated with Ar^8+^ ions of energy 1.75 MeV/a.u. and various fluences. Dotted lines show linear approximations to the corresponding sections of the experimental plots.

**Figure 3:**
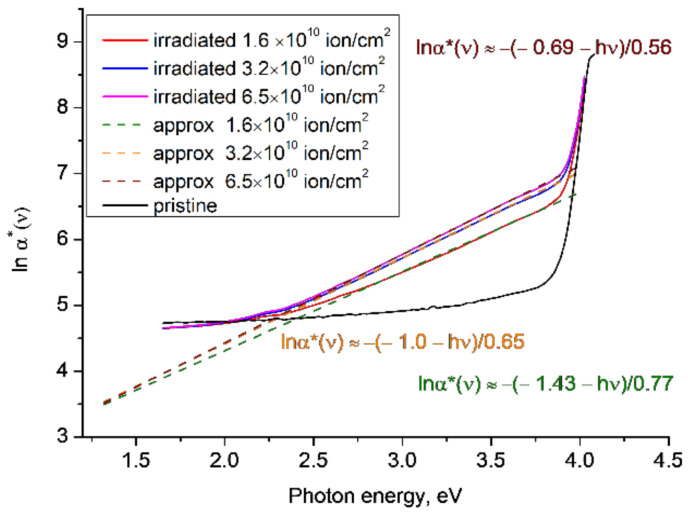
The functions lnα*(*ν*) for the experimental results reported in [12] for PET film irradiated with Kr^15+^ ions of energy 1.75 MeV/a.u. and various fluences. Dotted lines show linear approximations to the corresponding sections of the experimental plots.

**Figure 4:**
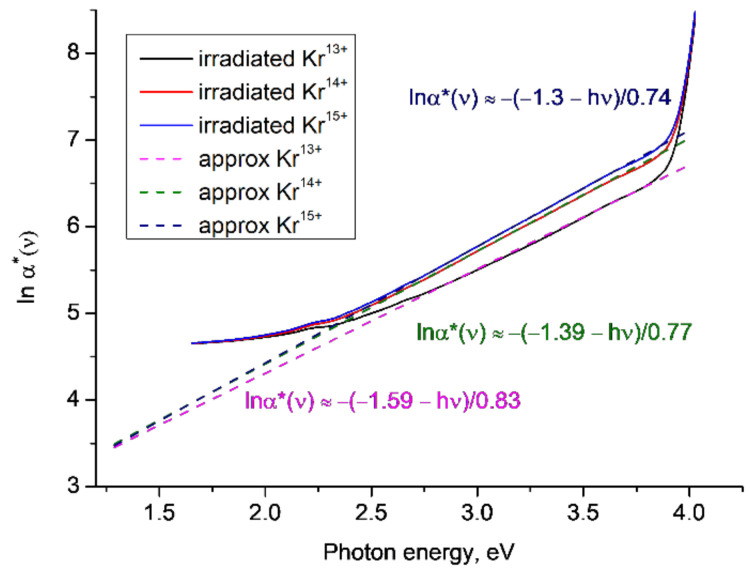
Functions lnα*(*ν*) for the experimental results reported [12] for PET film irradiated in oblique geometry with Kr ions of energy 1.2 MeV/a.u. with different charges and a fluence of 1 × 10^11^ cm^−2^. The dotted lines show linear approximations to the corresponding sections of the experimental plots.

**Figure 5:**
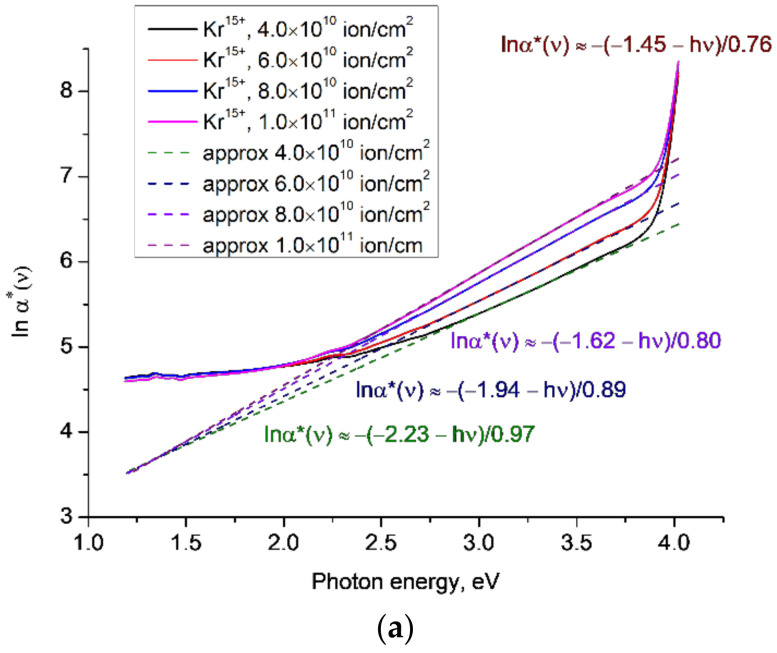

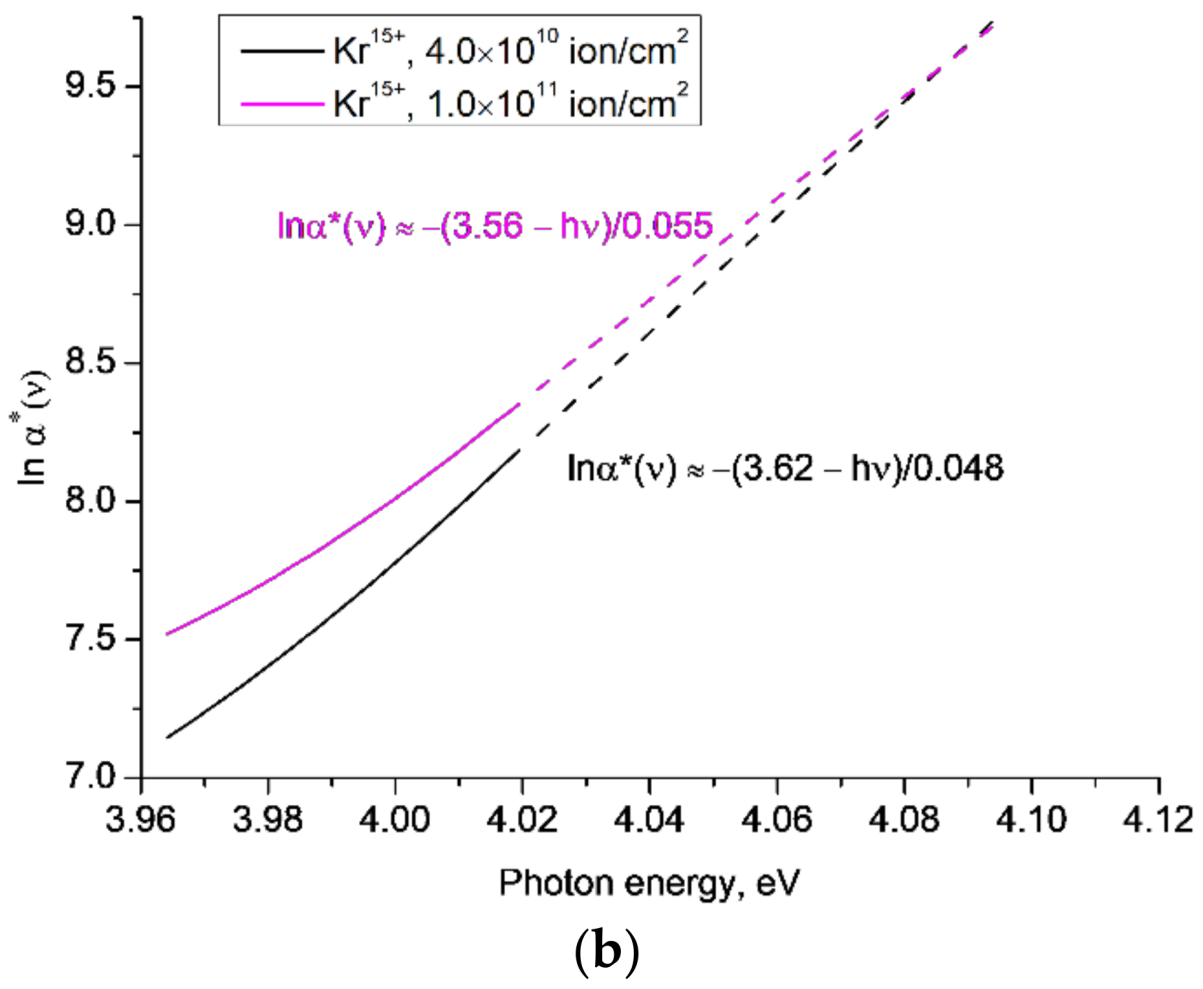
The functions lnα*(*ν*) for the experimental results reported in [21] for PET film samples irradiated with Kr^15+^ ions of energy 1.75 MeV/a.u. and various fluences: (**a**) general view; (**b**) enlarged view of the UV part of the spectrum around 4 eV. The dotted lines show linear approximations to the corresponding sections of the experimental plots.

In Figure 2, Figure 3, Figure 4 and Figure 5 below, we further explore the behaviour of lnα*(*ν*) through analysis of our previous experimental data for a range of irradiating ions and fluences and both normal and oblique irradiating geometries, reported in [12,21].

Figure 2 shows lnα*(*ν*) for PET film samples irradiated with Ar^8+^ ions of energy 1.75 MeV/a.u. in the normal geometry and fluences of 2.25 × 10^10^ cm^−2^, 4.5 × 10^10^ cm^−2^, 6 × 10^11^ cm^−2^ and 5 × 10^12^ cm^−2^, which were the subject of study in [12]. Samples irradiated with fluences of 4.5 × 10^10^ cm^−2^ and higher exhibit well-pronounced Urbach edges in the region of photon energies ~2.3–3.6 eV, similar to the lower-energy Urbach edge seen in the irradiated sample in Figure 1. In the high-energy region, the curves of lnα*(*ν*) show some tendency to converge around 4 eV, but the coarse spectral resolution of 1 nm used to collect this data and the above-mentioned limit *α*(*ν*)*d* < *5* for scattering to be negligible mean that there are insufficient data points to draw any conclusions about the position of the focal point. We note that in Figure 2 the approximating straight lines for the lower energy Urbach edge converge at a focal point at about 1.4–1.5 eV, which is where features were previously found in the transmission spectra of pristine and irradiated PET films [21,22] that we interpreted as direct exciton transitions at energies of 1.49 eV and 1.34 eV.

The gradient of the lines for the lower energy Urbach edge in Figure 2 increases with increasing fluence, while for the higher energy Urbach edge shown in Figure 1b, the trend is reversed. According to [31], the trend in the lower energy region is a consequence of the magnitude of the electric field in this region decreasing with increasing fluence. Since a well-formed focal point in Figure 2 is only observed for fluences over 4.5 × 10^10^ cm^−2^, which is when overlapping of latent tracks is clearly seen in the optical spectra [21], we suggest that the reduction in the magnitude of the electric field is due to the overlapping of the peripheral halos of the latent tracks [12,21,22,23,24,25].

These overlapping, statistically evenly distributed halos form a single area of electronically depleted material, which is characterized by the family of linear dependences of lnα*(*ν*) for photon energies of 2.2–3.5 eV. By analogy with [28], we suggest that the bandgap energy of the merged halo material is given by the low-energy focal point convergence in Figure 2, so is in the energy range of 1.4–1.5 eV. For ease of reference going forward, we will call these two convergences the lower and upper focal points.

Figure 3 plots lnα*(*ν*) for the experimental results reported in [12] for PET samples irradiated with Kr^15+^ ions of energy of 1.75 MeV/a.u. in the normal geometry and fluences of 1.6 × 10^10^ cm^−2^, 3.2 × 10^10^ cm^−2^ and 6.5 × 10^10^ cm^−2^. The behaviour is similar to that seen in Figure 2, with the gradient of the linear approximations of lnα*(*ν*) in the energy range ~2.2–3.7 eV increasing with increasing fluence and converging at a lower focal point at ~1.3 eV. The functions lnα*(*ν*) show some tendency to converge around 4 eV, but for the same reasons as Figure 2, there are insufficient experimental data points to draw any conclusions about the position of the upper focal point.

Figure 4 shows lnα*(*ν*) for samples of PET film irradiated with Kr ions of energy of 1.2 MeV/a.u. and a fluence of 1 × 10^11^ cm^−2^, for three different charges (13+, 14+ and 15+) and in the oblique geometry as described and reported in [12]. We again find that the linear approximations for lnα*(*ν*) in the Urbach edge region between ~2.2–3.7eV converge at a lower focal point at about 1.3 eV, although we note that this result is less reliable than previous cases, due to the small difference in the gradients of the lines. The gradient of the linear approximations of lnα*(*ν*) increases (i.e., the characteristic Urbach energy *W* decreases) with increasing ion charge, since higher SHI charges produce higher residual fields in their latent tracks.

Figure 5 plots lnα*(*ν*) for the experimental results reported in [21] for PET samples irradiated with ions of Kr^15+^ and energy of 1.75 MeV/a.u. for various fluences, where we used a higher resolution of 0.1 nm than in [12]. As for our earlier results, there is a clear lower focal point at around ~1.3 eV, an upper focal point at ~4.1 eV, and an increase in gradient of the linear approximations to lnα*(*ν*) in the lower-energy Urbach edge region for increasing fluences.

These results taken together show that the position of the focal points is largely independent of SHI charge and fluence, but the gradient and hence the value of the characteristic Urbach energy *W* is sensitive to both fluence and charge. The reasons for this sensitivity deserve a separate study, which we intend to conduct in the near future.

The values of the characteristic Urbach energy found from all the above linear approximations of lnα*(*ν*) in the energy range 2.3–3.6 eV are summarized in Table 1.

Table 1 shows that all values of the difference between the energy of the excited singlet state *S*_1_ and the Urbach energy *W* lie between the *T*_1_ = 3 eV and *T*_3_ = 3.7 eV triplet levels in PET film [39]. We believe that this indicates that under SHI irradiation the triplet levels broaden to form one wide band and *W* is the energy of a nonradiative transition from the excited state *S*_1_ to this triplet band. It can be seen from the three datasets (1, 2, 3) that, for a given experimental set-up and ion charge/energy, *W* decreases as the fluence increases. Following [31], we have interpreted this as due to a decrease in the magnitude of the residual electric field as the fluence increases and the latent track halos overlap. Dataset 4 shows that *W* also decreases as the ion charge increases. This inverse relationship cannot be explained in the *δ*-electron model, but can be understood in terms of the electron depletion of the polymer film material in the peripheral halos of the latent tracks. The dependence of *W* on the irradiating ion charge provides independent confirmation of the dependence of the post-irradiation molecular structure on the irradiating ion charge found in [25].

## 4. Discussion

These results clearly show the presence of two distinct materials in irradiated PET films, each with its own Urbach edge region and focal point associated with its bandgap energy. The existence of two regions of differing properties in SHI irradiated films is well-known and widely recognized as being due to the redistribution of electrons in the irradiated film under the influence of the ion electric field. Although some have argued that the inner core is left depleted of electrons by the kinematic action of swift *δ*-electrons [45], we have previously made the case that the effects of slow electrons [20] must also be considered. In the regimes under study, slow electrons (with energies below the atomic ionisation potentials in the medium) are quantitatively predominant, and move towards the track axis during the passage of a SHI, leading to a general electron enrichment of the track core and depletion of the outer halo [12,21,22,23,24,25]. The electret nature of the PET film means that the source of these slow electrons are the numerous intra- and inter-molecular traps with depths from 0.2 eV to 4.1 eV [26,38,39,40] that are present in PET, and not atomic electrons from the PET polymer chains. The passage of an ion through the pristine film pulls electrons from traps in the periphery into traps in the core, creating a radial distribution of emptied traps in the peripheral halo, with only the shallowest emptied at the outer edge and progressively deeper traps emptied closer to the track axis. Within the small core, the capacity of shallow traps is rapidly exceeded so that most electrons have to occupy deeper traps [26]. As the fluence increases, statistically the peripheral halos increasingly overlap, eventually forming a single region of electron-deficient material in which shallow traps are empty of electrons, as they have moved under the influence of the ion electric field into deep traps in the electron-enriched cores of the latent tracks. The emergence of two distinct regions within irradiated PET film explains the emergence of a second Urbach edge, and the presence of both an upper and lower focal point.

**Figure 6:**
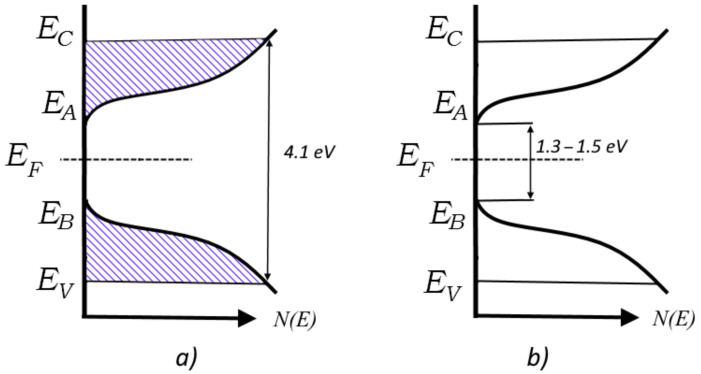
Schematic representation of density of states in PET film irradiated with SHI: (**a**) core of latent track; and (**b**) peripheral halo of latent track.

The position of these focal points can be understood by reference to the well-known Mott model of the density of states with a true bandgap, applicable to amorphous semiconductors and dielectrics that are transparent in the visible and IR parts of the spectrum [28], such as the PET film under study. This is depicted in Figure 6, where E_F_ is the Fermi level, E_C_ and E_V_ denote the energy levels that separate localised states from nonlocalized ones in the conduction band and valence band, and E_A_ and E_B_ are the boundaries of the tails of the conduction band and valence band, respectively. Shading shows areas where all localised states are full, and therefore unavailable due to the Pauli principle.

Figure 6a shows the situation where all shallow traps are occupied, leading to a bandgap of 4.1 eV, as measured in [38,39]. Figure 6b shows the situation when the shallow traps have been emptied, resulting in a bandgap of ~1.3–1.5 eV. Irradiation of PET film with highly charged very heavy ions (e.g., U, Xe, Bi) could shed light on whether this bandgap value characterizes the intrinsic properties of PET films, or just reflects the extent of trap emptying possible with the SHI charges and fluences used in these experiments.

We now return to the question formulated in [24] as to whether it is helpful at high irradiating fluences to continue to speak about overlapping of individual latent tracks. The evidence presented here concerning the structure of PET films when latent tracks are overlapping leads us to conclude that it may be more useful to consider such films as consisting of two materials. The electron-depleted halo region corresponds to a *p*-doped semiconductor with a bandgap of 1.3–1.5 eV, in which sit cylindrical electron-rich track cores, corresponding to an *n*-doped semiconductor with a bandgap of 4.1 eV. The boundaries between these two differently doped polymer materials thus correspond to conventional semiconductor *p-n* junctions.

The process of SHI irradiation also has parallels with chemical redox reactions, in which an oxidizing agent acquires electrons from the oxidized substance, thereby being reduced itself. This suggests that the electron-depleted halo might have similarities with oxidized materials (such as becoming more difficult to etch than pristine film), while the core might be expected to exhibit properties of reduced materials. The excess electrons in the core are trapped in its structure, rather than held in atomic bonds, as is the case with chemically reduced materials. They can be freed by the destruction of these structural traps, and it is well-known that free electrons are efficient catalysts [46]. This suggests that in alkali etching of irradiated films, the alkali acts as an initiation agent, destroying traps in the core and triggering electron catalysis that vastly speeds up the fragmentation of the PET chain molecules in the latent track cores. We believe that this electron catalysis in the cores explains the huge difference in the etching rates of irradiated PET films along and across the direction of the irradiating ion paths [47].

## 5. Conclusions

Our analysis shows that the ‘nontransmittance’ functions a*(*ν*) for PET film irradiated with various fluences of SHI have an exponential form in two photon energy intervals within the region of the absorption edge—so exhibiting two Urbach edges. In semi-logarithmic coordinates, for each PET film sample and each of the two Urbach edges, the set of linear approximations to lnα*(*ν*) for different SHI irradiating fluences form families of straight lines showing a fan-shaped rotation with increasing fluence. The upper Urbach edge family of lines in the UV region has a focal point at 4.1 eV, associated with the electron-enriched inner core of the latent track. The linear approximations in the lower Urbach edge region, located mainly in the visible part of the spectrum, have a focal point in the range of 1.3–1.5 eV, associated with the electron-depleted peripheral halo of the latent track. The characteristic Urbach energy *W* for the lower energy Urbach edge in all our experimental data sets lies within the range of the energy of a nonradiative transition from the excited singlet state to triplet band of the benzene-carboxyl complexes of repeat units of chain molecules of PET film. The inner electron-enriched core and outer electron-depleted halo of latent tracks in the PET film are analogous to *n*- and *p*-doped semiconductors, respectively, and the boundary between the regions corresponds to a conventional semiconductor *p-n* junction. The excess electrons in the core of the latent track, which are held in structural traps rather than chemical bonds, can act as a catalyst when their release is triggered by alkali etching, vastly accelerating the fragmentation of PET chain molecules along the SHI pathway.

## Figures and Tables

**Table 1:** Experimentally determined characteristic Urbach energy values for PET films after SHI irradiation.

Set	Article	Irradiation Conditions	Value of *W*, eV	Value of (*S*_1_-*W*), eV
1	[12]	Ar^8+^; 1.75 MeV/a.u.; normal; 4.5 × 10^10^ cm^−2^	0.68	3.42
		Ar^8+^; 1.75 MeV/a.u.; normal; 6 × 10^11^ cm^−2^	0.54	3.56
		Ar^8+^; 1.75 MeV/a.u.; normal; 5 × 10^12^ cm^−2^	0.43	3.67
2	[21]	Kr^15+^; 1.75 MeV/a.u.; cylinder; 4 × 10^10^ cm^−2^	0.97	3.13
		Kr^15+^; 1.75 MeV/a.u.; cylinder; 6 × 10^10^ cm^−2^	0.89	3.21
		Kr^15+^; 1.75 MeV/a.u.; cylinder; 8 × 10^10^ cm^−2^	0.8	3.3
		Kr^15+^; 1.75 MeV/a.u.; cylinder; 1 × 10^11^ cm^−2^	0.76	3.34
3	[22]	Kr^15+^; 1.75 MeV/a.u.; normal; 1.9 × 10^10^ cm^−2^	0.74	3.36
	[12]	Kr^15+^; 1.75 MeV/a.u.; normal; 1.6 × 10^10^ cm^−2^	0.77	3.33
		Kr^15+^; 1.75 MeV/a.u.; normal; 3.2 × 10^10^ cm^−2^	0.65	3.45
		Kr^15+^; 1.75 MeV/a.u.; normal; 6.5 × 10^10^ cm^−2^	0.56	3.54
4	[12]	Kr^13+^; 1.2 MeV/a.u.; oblique; 1 × 10^11^ cm^−2^	0.83	3.27
		Kr^14+^; 1.2 MeV/a.u.; oblique; 1 × 10^11^ cm^−2^	0.77	3.33
		Kr^15+^; 1.2 MeV/a.u.; oblique; 1 × 10^11^ cm^−2^	0.74	3.36

## Data Availability

Not applicable.
